# The application of artificial intelligence technology in education influences Chinese adolescent’s emotional perception

**DOI:** 10.1007/s12144-023-04727-6

**Published:** 2023-05-11

**Authors:** Tinghong Lai, Xianqin Zeng, Bin Xu, Chuyin Xie, Yanxiu Liu, Zheng Wang, Hong Lu, Shimin Fu

**Affiliations:** 1 Center for Brain and Cognitive Science, School of Education, Guangzhou University, Guangzhou, China; 2 Gannan Medical University, Ganzhou, China; 3 Experimental Center (Education) of National Intelligent Society Governance , Student Development Center Under Intelligent Education, Guangzhou, China; 4 Management Center for Quality Education of Baiyun District, Guangzhou, China

**Keywords:** Artificial intelligence, Technology, Education, Chinese adolescents, Emotional perception, Influence

## Abstract

Humans need to accurately infer the intentions and feelings of others to engage in successful social interaction. However, the application of artificial intelligence technology in Education (AIEd) forms a human–machine collaborative environment which changed the interaction relationship of individuals, it may have an affect on them. This study aimed to explore whether AIEd affects adolescents’ emotional perception. Combined with the actual teaching situation and the result of the questionnaire, 1332 students recruited through random sampling from AI Curriculum Reform Demonstration Schools in Guangzhou participated in this study. Different emotional priming stimulative materials (sentences and situational pictures) were used in the experiments. The task was designed to investigate adolescents’ reaction time to emotional faces (positive, negative). After eliminating blank data and invalid data with response time greater than 150 ms, 977 and 962 valid data were included in the statistical analysis in experiment 1 and experiment 2 respectively. Results show that AIEd has a negative effect on adolescents’ emotional perception. Prior research has focused on theory to the exclusion of practical applications and the psychological impact of AIEd, thus this study makes an innovative contribution in exploring the impact of the application of artificial intelligence technology in education on adolescents’ physical and mental development by using empirical research methods.

## Introduction

Artificial intelligence (AI) is widely used in the field of education at present, and the application of artificial intelligence technology in education (AIEd) is a new trend in educational innovation and development. Particularly after the outbreak of COVID-19 in 2020, large-scale online teaching has become a big test of how AI technology might enable education. But people know little about AIEd’s possible impacts, especially on the physical and mental development of the educated. Adolescents are the principal recipients of AIEd, and they are in a critical period in which very easily affected by the external environment (Chiang et al., 2020; Lu et al., 2020). It is important to explore the possible impacts of AIEd in order to avoid the possible adverse effects. However, previous studies have only discussed the influence of AIEd on adolescent at the theoretical level and the empirical research was relatively lacking, the lack of research into emotion and influencing factors research has always been a prominent problem in AIEd (Chen & Zhang, 2019). So, it is necessary to pay attention to the impact of AIEd on adolescents’ physical and mental.

## Application of artificial intelligence technology in education

In recent years, the application of artificial intelligence technology in education (AIEd) has become a hot topic, such as machine learning, E-learning, MOOC (Massive Open Online Course) and all. AIEd can be understood as integrating artificial intelligence (AI) technology into the scenes of education (Wu, et al., 2017). At present, a number of key AI technologies including machine learning, knowledge mapping, and natural language processing are gradually being applied in education. In general, there are five typical ways in which AIEd is applied: an intelligent education environment, intelligent learning process support, intelligent educational evaluation, intelligent teacher assistance, and intelligent educational management and services (Lu, et al., 2021). In this study, AIEd refers to the universal application of AI technology in education, i.e., the new technologies be used to improve teaching methods and enhance learning efficiency, expand teaching time–space environment, and improve teaching management and services. These can also be referred to as VR teaching, online teaching, flat panel teaching, etc. Research to date has shown there are three main forms of AIEd being used in the Artificial Intelligence Curriculum Reform Experimental Schools in Guangzhou. One is the form of curriculum teaching, such as information technology courses, general technology courses, flat panel teaching, intelligent reading, etc. The second takes the form of interest classes, such as programming courses, assembling robots, etc. The third involves mass organization, such as 3D printing, Leo plug-ins, teaching box, etc.

## Impact of the application of artificial intelligence technology in education on individual

Previous studies have shown that the application of artificial intelligence (AI) technology is conducive to individual development. For example, children who interact with robots show a high level of creativity (Ali et al., 2020), and wearable machines can enhance the expression ability of adolescents with autism spectrum disorder (Xiao et al., 2020). However, some studies have shown that the application of artificial intelligence technology is disadvantageous to individual development. For example, frequent use of intelligent electronic devices has a negative impact on adolescents’ interpersonal relationships (Halpern & Katz, 2017) and social adaptability (Jin et al., 2017), and the elderly who are cared for by robot partners feel more lonely and emotionally indifferent (Thibault et al., 2019).

The application of artificial intelligence technology in education (AIEd) is based on the Internet and computer, it forms a man–machine collaborative environment in which different electronic or intelligent devices become the intermediary connecting students and teachers with social interaction changed from “two-dimensional” to “three-dimensional” (Xiao & Hu, 2010). Some researchers believe that AIEd brings more opportunities than threats (Holmes et al., 2019; Hwang et al., 2020), in that it is more effective and has a more positive influence on students’ academic achievement and performance compared to traditional learning tools and environments (Ma et al., 2014; Erdemir et al., 2015). For example, VR (virtual reality) teaching, which is based on AI technology has been found to have a positive impact in significantly improving students’ performance (Li et al., 2019). However, AIEd which changed the interpersonal interaction in education may also have negative impact, as it leads to social interactional isolation and weaken interpersonal relationships (Li & Wang, 2018) and the sense of social presence (Zhang, et al., 2019; Diemer et al., 2015) between educators and educated. Research demonstrated that 82% of information in teaching is transmitted through nonverbal communication, nonverbal intimate behaviors (such as facing students, smiling, approaching students, eye contact and communication, voice cadence), and positive posture are the center of effective teaching (Frymier et al., 1996). The more nonverbal intimate behaviors, the better the effect on students’ emotional learning (Pogue & Ahyun, 2006). AIEd reduces the nonverbal intimacy behaviors between teachers and students, and thus their sense of social presence and interpersonal interaction is weakened. Some researchers believe that there are potential risks of AIEd. For example, although students increased their sense of presence by enhancing attention to the VR teaching environment, but it may weaken the real social interaction (Diemer et al., 2015). Using intelligent technology to collect students’ learning data may cause safety and ethical problem due to data leakage, because students may be “labeled” as problem student or student with learning difficulties that will influence his/her psychology (Ferguson et al., 2016; Potgieter, 2020). Some researchers are even worried that AIEd may deviate from the purpose of education and become a potential educational risk due to the bias of its designers and executants (Zanetti, 2019).

## Rationale and hypothesis

Emotional perception (EP) is an ability to clearly perceive the emotions of oneself and others and adjust their behaviors accordingly in social situations (Salovey & Mayer, 1989), it includes interpersonal skills (Gardner, 1993). According to the theory of social presence and the theory of social cue reduction which are based on cue filtering orientation, media communication is more prone than face-to-face communication to weaken the ability and expectation of individual to establish social interaction due to the lack of important nonverbal and situational cues, such as those involving vision, hearing and touch (Sprecher, 2014). Although non-face-to-face online social contact produces less social pressure and lower social anxiety than real face-to-face social contact, most young people with social anxiety further escape from real social contact after obtaining social support through online (He et al., 2014), it is disadvantageous to them. The application of artificial intelligence technology in education (AIEd) is based on computers and other media technologies, making it inseparable from the use of intelligent devices such as the Internet and electronic or intelligent equipment. However, education is a kind of social activity, and interaction and cooperation are the core of the teaching process. AIEd makes machines become the intermediary connecting students and teachers, which changes the interpersonal relationship of teaching from human–human to human–machine-human. The changed space–time relationship of teaching leads to a decrease in real teacher-student interpersonal interaction, and the students’ sense of social presence is weakened. In addition, AIEd has great situational difference from conventional teaching and lacks of sufficient nonverbal clues, situational clues and other important information which will lead to a reduction in adolescents’ social involvement, thereby increasing social isolation and social anxiety. When adolescents stay in the AIEd environment for a long time, their interpersonal skills may be affected by the lack of real social interaction.

Based on above analysis, we hypothesized that AIEd affects adolescents’ emotional perception, i.e., if the difference in average reaction time between the AI group and non-AI group is significant, that suggests AIEd affects adolescents’ emotional perception; Specifically, if the average reaction time of the AI group is significantly slower than that of the non-AI group, that suggests AIEd has a negative impact on adolescents’ emotional perception, and vice versa. Because emotional perception affected by different causes (semantic and visual), different stimulation materials were used to prime individuals’ emotions to the same valence level in the study, and then the difference in average reaction time of individuals between the AI group and non-AI group was analyzed in terms of the emotional valence of the target stimulation. As AIEd refers to the deep integration of artificial intelligence technology and education, so the distinction between the AI group and non-AI group in this study was based on whether AI technologies were used in the process of education, such as VR teaching, online teaching, flat panel teaching, etc.

## Experiment 1

### Materials and methods

#### Participants

1332 students recruited through random sampling from classes in different grades of AI Curriculum Reform Demonstration Schools in Guangzhou participated in this study. Participants included 342 primary school students (*M*_year_ = 10.6), 351 junior high school students (*M*_year_ = 13.1) and 639 senior high school students (*M*_year_ = 15.8). All participants had normal hearing and normal or corrected-to-normal vision. This study was approved by the Ethics Committee of Guangzhou University (Protocol number: GZHU2020010). All participants and their guardians had signed written informed consent.

The Mobile Phone Usage Questionnaire was used to investigate the usage of AI among adolescents. One of the items, “*Are you using AI / smart phones, tablets and other intelligent devices to learning*”, is scored with options 1 = *yes* and 2 = *no*. Based on their response and the actual teaching situation, students were divided into the AI and non-AI group.

#### Stimuli and procedure

The experiment was implemented using E-Prime software (version 2.0, Psychology Software Tools, Inc., Pittsburgh, USA). Twelve sentences (six positive, six negative) taken from previous studies were used as emotional priming stimuli (see Table 1), and twelve emotional faces, selected from the Chinese Facial Affective Picture System (CFAPS), were used as targets.

**Table 1:** Twelve sentences in Experiment 1 (used as emotional priming stimuli)

Positive Sentences	Negative Sentences
I win the first in the college entrance examination	Mother is critically ill
My short video got one million likes	Her five years old son died of illness
I won the first place in the table tennis match	The child was abducted
My essay was selected as a model to read	The dancer was paralyzed in a car accident
I was chosen as the school flag bearer	Grandpa, who was dependent on each other since childhood, died
I received a college admission notice	His daughter died accidentally on her way home from school

Participants were tested individually in a quiet computer room. The main tester explained the precautions and emphasized the need to be quiet and not quit partway through the procedure as far as possible. The formal experiment consisted of 12 experimental blocks, each containing 12 trials. The directions were presented, and participants pressed any key to begin the formal experiment after they understood the task. A fixation point was presented for 500 ms in the center screen, then a sentence for 2500 ms (such as my essay was selected as a model to read) randomized, and then finally an emotional face was presented for 1000 ms randomized. The task was to judge the facial emotion as quickly and accurately as possible. Participants were instructed to press F if the facial emotion was positive and J if it was negative. The schematic diagram of the experiment is as follows:
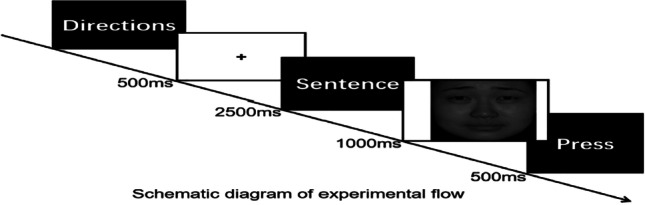


Before the formal experiment, participants completed 24 practice trials. In the practice trials, participants were given feedback (correct, incorrect, or no response) on their judgment of emotional responses, so they could understand the task better. In the formal experiment, there was no feedback on whether the emotional response was correct or not. In this study, we analyze the accuracy of participants’ emotional responses. The results show that the accuracy is nearly 75%, indicating that the procedure was effective and could be further analyzed. As there is relative lack of research on the impact of AIEd on adolescents’ physical and mental development, it is unclear at present whether the correctness of emotional response is a relevant factor or not. Moreover, this study is exploratory research, so we only consider cases when the accuracy rate of emotional response reaches a certain proportion, and do not consider the situation of incorrect emotional responses, which can be further explored in the future.

## Results

We analyzed the difference of average reaction time to facial emotion between AI group and non-AI group. Invalid data with response times faster than 150 ms or blank were eliminated.

We conducted an independent sample t-test and found that there was significant difference in emotional perception between the AI group and the non-AI group (*t* (975) = 2.18, *p* < 0.05, *Cohen’s d* = 0.18). The average reaction time of the AI group (*M* ± *SD* = 626.51 ± 112.78) was significantly slower than that of the non-AI group (*M* ± *SD* = 606.16 ± 127.90), indicating that AIEd affects adolescents’ emotional perception negatively. This result meets our experimental expectation. See Table 2.

**Table 2:** Results of differences in emotional perception between AI group and non-AI group in Experiment 1

	Emotional perception				
	*N*	*M*	*SD*	*t*	*p*
AI group	785	626.51	112.77	2.18*	0.029
Non-AI group	192	606.16	127.91		

* = *p* < 0.05, ** = *p* < 0.01, *** = *p* < 0.001

**Fig. 1:**
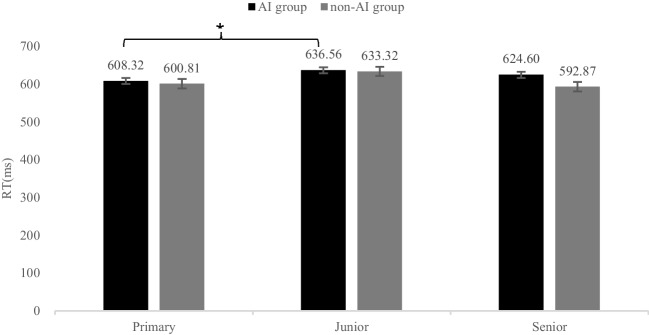
Comparison results of average reaction time between AI group and non- AI group in different grades in Experiment 1. *Note: *^⁎^ = *p* < 0.05, ** = *p* < 0.01, *** = *p* < 0.001

Results of two-way ANOVA with average reaction time as the dependent variable, and grade and group as independent variables showed that the main effect of the grade was significant (*F* (2, 976) = 3.67,* p* < 0.05, ƞ_p_^2^ = 0.07). The result of post hoc multiple comparative analysis of grades found that the main effect difference of grades come from primary and junior middle school. The primary school students reacted fastest (*M* ± *SD* = 604 ± 12.55), while the junior middle school students reacted slowest (*M* ± *SD* = 634.94 ± 8.33). The main effect of the group was not significant (*F* (1, 976) = 1.70,* p* > 0.05, ƞ_p_^2^ = 0.02), although the average reaction time of AI group (*M* ± *SD* = 623.16 ± 4.69) was slower than that of non-AI group (*M* ± *SD* = 608.99 ± 9.82). The interaction between grade and group was not significant (*F* (2, 976) = 1.06,* p* > 0.05, ƞ_p_^2^ = 0.02). See Fig. 1 and Table 3.

**Table 3:** Post hoc multiple comparison results of grades in Experiment 1

Grade	Grade	*SD*	*Sig*
Primary	Junior	-29.07*	0.033
	Senior	-10.76	0.593
Junior	Primary	29.07*	0.033
	Senior	18.32	0.063
Senior	Primary	10.76	0.593
	Junior	-18.32	0.630

* = *p* < 0.05, ** = *p* < 0.01, *** = *p* < 0.001

Prior studies have demonstrated that facial expressions (Yuki et al., 2007), vocal tone, and verbal meaning (Ishii et al., 2003; Kitayama & Ishill, 2002) are all cues for emotional perception. They have also shown that preschoolers can match expressions with emotion words for some emotions such as happiness, anger, and sadness (Bullock & Russell, 1984; Harrigan, 1984; Reichenbach & Masters, 1983; Russell & Widen, 2002). With the increase of age, the weight of visual information in the process of emotional perception increases (Kawahara et al., 2021). Because the emotional priming stimuli were sentences in experiment 1, it may be the case that due to age, learning basis, or lack of comprehension, there were understanding differences affected the effect of emotional priming. We therefore considered changing sentences into situational pictures (positive, negative) to eliminate the possible influence of the nature of the materials in experiment 2.

## Experiment 2

### Participants, materials, and procedure

The participants, paradigm, procedure and the response accuracy analysis were identical to those in Experiment 1. Twelve situational pictures (six positive, six negative) from the Internet, with emotional valences estimated by students who didn’t participate in the experiment, were used (see Table 4).

**Table 4:**
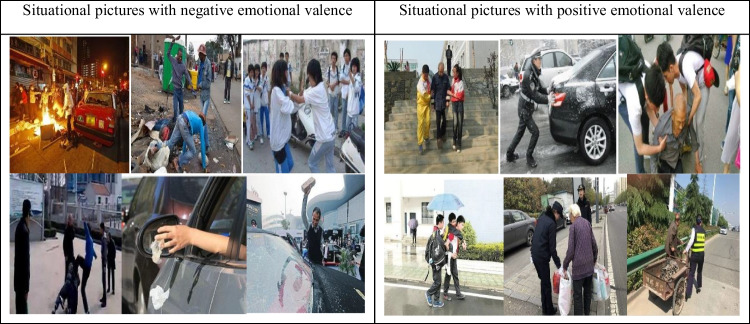
Situational pictures in Experiment 2 (estimated by students who didn’t participate)

Emotional faces were the same as those used in Experiment 1. The formal experiment consisted of 12 experimental blocks, each containing 12 trials. The directions were presented first, and when the participants felt they understood, they pressed any key to begin the formal experiment. A fixation was presented for 500 ms, followed by a situational picture for 1000 ms (such as a depiction of someone helping others) randomized, then finally an emotional face for 1000 ms randomized. The task was to judge facial emotion as quickly and accuracy as possible. Participants were instructed to press F if the facial emotion was positive and J if it was negative. The schematic diagram is as follows:
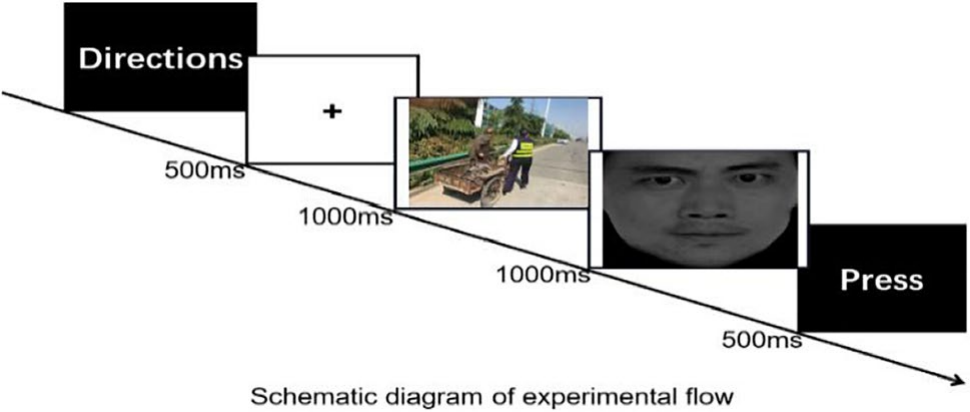


## Results

The invalid data were eliminated just as in Experiment 1. Results of the independent-sample t-test showed that the difference in emotional perception between AI group and non-AI group was significant (*t* (960) = 3.43, *p* < 0.001, *Cohen’s d* = 0.29) (See Table 5). The average reaction time of the AI group (*M* ± *SD* = 657.19 ± 131.58) was significantly slower than that of non- AI group (*M* ± *SD* = 618.03 ± 159.16), indicating that AIEd affects adolescents’ emotional perception negatively. It was consistent with the result of Experiment 1.

**Table 5:** Results of differences in emotional perception between AI group and non-AI group in Experiment 2

	Emotional perception				
	*N*	*M*	*SD*	*t*	*p*
AI group	786	657.19	131.58	3.43***	0.000
Non-AI group	176	618.03	159.16		

* = *p* < 0.05, ** = *p* < 0.01, *** = *p* < 0.001

**Fig. 2:**
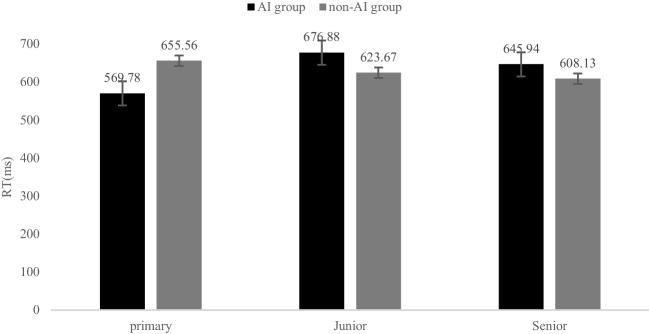
Comparison results of average reaction time between AI group and non- AI group in different grades in Experiment 2

**Fig. 3:**
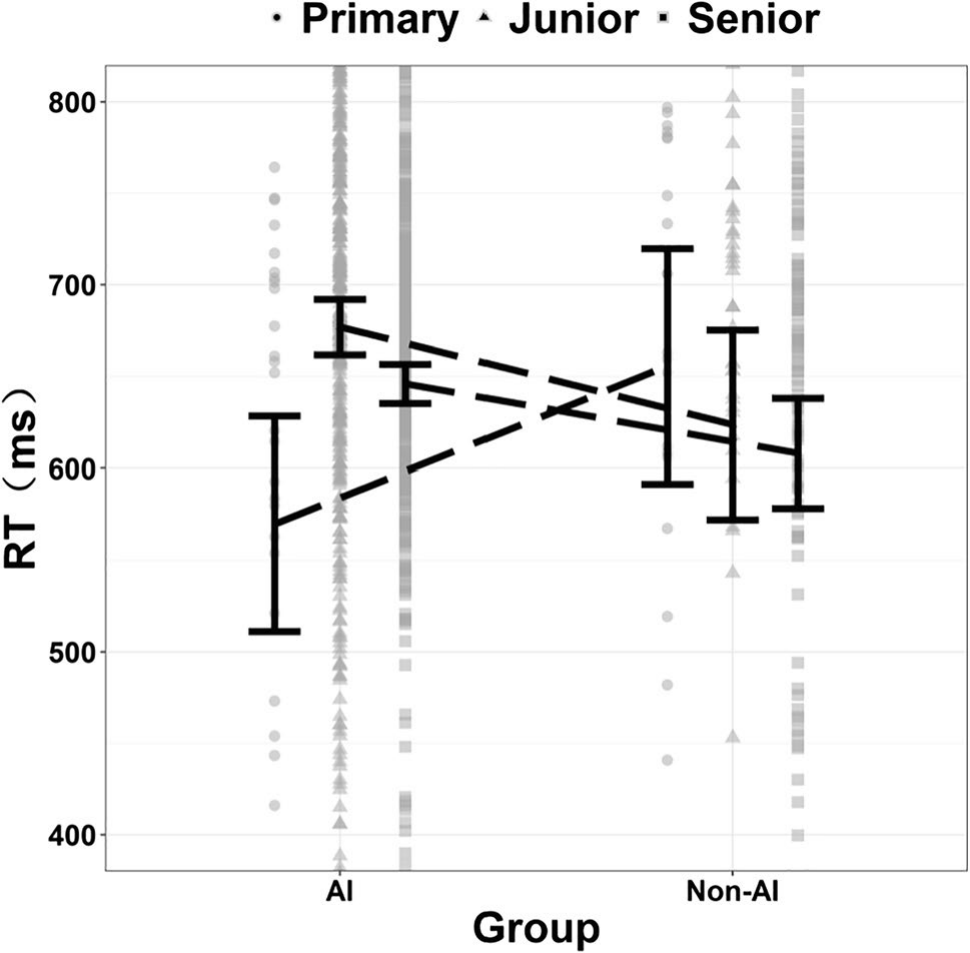
The Interaction between grade and group in Experiment 2

Results of two-way ANOVA with average reaction time as dependent variable, and grade and group as independent variables showed that the main effect of grade (*F* (2, 961) = 2.16, *p* > 0.05, ƞ_p_^2^ = 0.04) was not significant, the average reaction time of primary school students (*M* ± *SD* = 612.67 ± 18.40) was fastest and the average reaction time of junior middle school students was slowest (*M* ± *SD* = 650.27 ± 11.03). The main effect of group (*F* (1, 961) = 0.01, *p* > 0.05, ƞ_p_^2^ = 0.00) was not significant, even the average reaction time of AI group (*M* ± *SD* = 630.87 ± 8.52) was slower than that of non-AI group (*M* ± *SD* = 629.12 ± 12.47). The interaction between grade and group was significant (*F* (2, 961) = 5.65, *p* < 0.01, ƞ_p_^2^ = 0.12). See Fig. 2 and 3.

## General discussion

In reality, educators should be able to capture the dynamics in the learning group whether in an online or an on-campus setting, and respond empathically and in a pedagogically meaningful way (Olaf et al., 2019). The application of artificial intelligence technology in education (AIEd) should retain human attributes in order to avoid the possible adverse effects of excessive use of technology. Results of this study indicated that AIEd has a negative impact on adolescents’ emotional perception. It may be the use of AI technology leads to real interpersonal interaction between educator and educated decreased, as well as relationship changed. AIEd changes the interaction mode and reduces nonverbal intimacy behaviors between teachers and students (Su and Bartel, 2019). The human–computer collaborative environment can easily lead to indifference to interpersonal relations and weaken the individual’s perception of others’ emotions, which affecting their emotional perception. Moreover, an over-emphasis on the role of technology neglects care and respect for adolescents which in turn affect adolescents’ capability for emotional perception.

When the priming stimuli are sentences (Experiment 1), the main effect of grades on emotional perception is significant, and the difference comes from primary school students and junior high school students. But there is no significant difference between senior high school students and other grades students. The reason for this result maybe that there are differences in the understanding of sentences between primary school students and junior high school students which result in different emotional priming states, then result in different judgments on target stimuli. Senior high school students’ judgment of target stimuli is not affected by the priming stimuli easily, so there is no significant difference between senior high school students and other grades students. When the priming stimuli are situational pictures (Experiment 2), the main effect of grades on emotional perception is not significant, there is no significant difference between grades. With the increase of age, the weight of visual information in the process of emotional perception increases (Kawahara et al., 2021), there is no significant difference in the understanding of visual information among students of different grades, the emotional priming states are consistent which not affect the judgment of target stimuli, therefore, there is no significant difference between grades.

## Limitation and future directions

In conclusion, several issues should be examined in future studies. First, this study only examined the correlation between AIEd and emotional perception, but could not determine their causal relationship. Future study could examine the causal relationship between AIEd and adolescents’ psychology, such as adopting tracking research design and EEG/ERP technologies. Second, this study did not consider other factors that may affect emotional perception, for example personality; these factors can be explored more comprehensively in the future. Third, students are not deeply involved as AIEd is in the initial stage in China at present. With the in-depth popularization of AIEd, more rigorous grouping methods of AI teaching can be used in the future research. Lastly, the sample maybe a little small in this study. Although it is sufficiently large to detect differences, it will be more objective and scientific to replicate these findings with a larger sample.

## Data Availability

Yes, but upon request.
